# A Hybrid Intelligent Diagnosis Approach for Quick Screening of Alzheimer's Disease Based on Multiple Neuropsychological Rating Scales

**DOI:** 10.1155/2015/258761

**Published:** 2015-03-01

**Authors:** Ziming Yin, Yinhong Zhao, Xudong Lu, Huilong Duan

**Affiliations:** ^1^College of Biomedical Engineering & Instrument Science, Zhejiang University, Hangzhou, Zhejiang 31002, China; ^2^China National Center for Biotechnology Development, Building D, No. 16, Xisihuanzhonglu, Haidian District, Beijing 100036, China

## Abstract

Neuropsychological testing is an effective means for the screening of Alzheimer's disease. Multiple neuropsychological rating scales should be used together to get subjects' comprehensive cognitive state due to the limitation of a single scale, but it is difficult to operate in primary clinical settings because of the inadequacy of time and qualified clinicians. Aiming at identifying AD's stages more accurately and conveniently in screening, we proposed a computer-aided diagnosis approach based on critical items extracted from multiple neuropsychological scales. The proposed hybrid intelligent approach combines the strengths of rough sets, genetic algorithm, and Bayesian network. There are two stages: one is attributes reduction technique based on rough sets and genetic algorithm, which can find out the most discriminative items for AD diagnosis in scales; the other is uncertain reasoning technique based on Bayesian network, which can forecast the probability of suffering from AD. The experimental data set consists of 500 cases collected by a top hospital in China and each case is determined by the expert panel. The results showed that the proposed approach could not only reduce items drastically with the same classification precision, but also perform better on identifying different stages of AD comparing with other existing scales.

## 1. Introduction

Alzheimer's disease (AD) is a degenerative senile dementia characterized by memory loss and cognitive functions disorders, and it is also one of the main types of senile dementia [1]. As AD has a slow onset and no highly specific diagnostic indicators at the early stage of the disease, it is particularly challenging for primary clinicians to identify transition points (from the asymptomatic phase to the symptomatic predementia phase to dementia onset) for individual patients [2, 3]. It is, nevertheless, important to identify these transition points between different stages, because studies [4] have proved that targeted therapies may help slow down the progress of the disease and improve quality of life for patients and their families.

**Figure 1:**
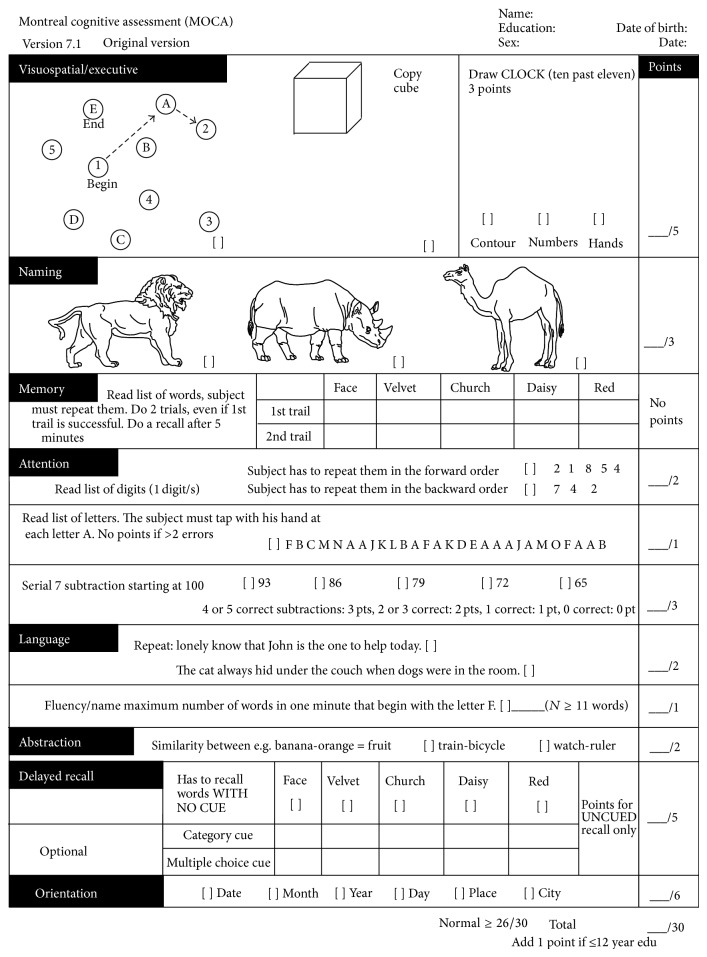
Montreal Cognitive Assessment (MOCA).

Due to the lack of advanced medical facilities (advanced imaging and cerebrospinal fluid measures), the screening of AD usually depends on the use of neuropsychological rating scales in primary clinics. Various neuropsychological rating scales, which are considered as a reliable and valid standardized testing tool, have been designed for cognitive abilities screening, and many of them have yielded good results as decision-making tools, such as minimental state examination (MMSE) [5], clinical dementia rating (CDR) [6], Montreal Cognitive Assessment (MoCA) [7], Geriatric Depression Scale (GDS) [8], and Activity of Daily Living Scale (ADL) [9].  Table 1 and Figure 1 are two most commonly used rating scales (the MMSE and the MoCA) in clinical practice.

However, each neuropsychological rating scale has its emphasis and limitation. A previous study has shown some scales do not perform well in one or more cognitive domains [10]. Multiple neuropsychological rating scales can cover more comprehensive cognitive domains. Therefore, multiple scales should be used together in order to get patients' comprehensive cognitive status, which can help doctors to make correct diagnosis. However, this will bring two challenges: (1) neuropsychological testing requires highly trained assessors [11], while most primary clinicians are not qualified to conduct a full mental status examination or interpret a battery of scales' score; it is difficult for them to offer exact judgments about the examinee's cognitive state [12]. (2) Neuropsychological testing is quiet time consuming; the elders cooperate well only for short periods with the limitation of vitality and cognition [13], so long-time testing will bring negative impact on the quality of neuropsychological testing. Thus, we can conclude that the screening of AD in primary clinics should be based on the criteria that can get maximum accuracy in a convenient way within limited time.

To solve the above-mentioned challenges, identifying the items with the best ability to distinguishing AD (called critical items for short) from a battery of commonly used rating scales may help improve the efficiency of cognitive abilities screening. Then, a well-performance decision-making model, while the previously selected items can be taken as its input, may help primary clinicians improve diagnostic accuracy in routine clinical practice. So in this paper, we suggest dealing with the screening of AD by means of a two-stage hybrid intelligent approach based on multineuropsychological rating scales analysis: in Stage 1, use a genetic algorithm-rough sets (GA-RS) model to identify critical items, and in Stage 2, use a Bayesian network to develop a diagnosis assisting model of AD based on the selected items. This hybrid intelligent technique takes the advantage of attributes reduction of rough set theory requiring no prior knowledge and the uncertain reasoning ability of Bayesian network to build a relatively convenient and accurate decision-making model for primary clinicians.

The rest of this paper is organized as follows: Section 2 introduces the related work; Section 3 introduces basic concepts behind rough set theory, genetic algorithm, and Bayesian network; Section 4 presents the proposed approach including the proposed GA-RS attributes reduction algorithm applied in AD and the Bayesian network model constructed for AD diagnosis; Section 5 describes the evaluation results of the proposed model; then in Section 6, the discussions on some benefits and limitations of the proposed approach in the clinical environment are made; Section 7 draws conclusions and future work.

## 2. Related Work

Since the application of multiple neuropsychological rating scales is very time consuming and challenging for primary care physicians, many researchers have been trying to find out the most effective screening method for clinical application. For example, some scholars have tried to simplify the MMSE, which is the most widely used scale for screening dementia. Lou et al. [14] reported a 16-item simplified version of the original MMSE with high sensitivity and specificity. Callahan et al. [15] designed a six-item screener, which derived from the MMSE, the Blessed Dementia Rating Scale (BDRS), and the Word List Recall. And its sensitivity and specificity for a diagnosis of dementia reached 88.7% and 88.0%. In addition to the study of the MMSE, some researchers also have studied the combination of multiple neuropsychological rating scales. For example, Chen et al. [16] proposed an eight-item test, subtracted from the MMSE, the Clock Drawing Test (CDT), and the Instrumental Activities of Daily Living Scale (IADL). The evaluation result revealed that it was a sufficient and simple tool for the screening of early dementia in primary care clinics. Besides, other studies [17–20] have reported various screening methods such as instruments designed for detection of memory, attention-executive function, visuospatial ability, and interview with reliable informants. After a general survey of the above studies, we found that all these researchers selected items according to their own clinical intuition regarding domains of impairment commonly encountered in AD rather than objective data analysis based on past cases. This selection was influenced by subjective factor easily. After selecting discriminative items, a large number of experiments need to be done for confirming weight coefficient of each item. For instance, the MoCA team spent over 5 years modifying the MoCA for clinical use [7]. So a method based on data mining may bring a new thought to searching the most discriminative items from multiple neuropsychological rating scales. It is based on large-scale objective clinical evidences, and, moreover, it can not only improve efficiency of identifying critical items but also adjust weight coefficient of items automatically.

The computer-aided diagnosis of AD is always the hot topic of research in the last few years. DeFigueiredo et al. [21] presented an algorithm based on the analysis of computer tomography image (CT) data from brain. This algorithm used an optimal interpolative neural network to classify individuals into four different groups (i.e., clinically diagnosed groups of elderly normal, demented, AD, and vascular dementia subjects). Ramírez et al. [22] showed a computer-aided diagnosis (CAD) system for the diagnosis of AD. His method is based on partial least squares regression model and a random forest predictor. The analyzed data is from single photon emission computed tomography (SPECT). Further works on SPECT data have reported high AD classification accuracy [23–25]. Duchesne et al. [26] presented their work on automated computer classification in Alzheimer's dementia using the context of cross-sectional analysis of magnetic resonance images (MRI). Daliri [27] also presented an automated method for diagnosing AD from brain MR images. In addition to volumetric MRI, diffusion tensor imaging (DTI) has increasingly been used to detect microstructural brain differences in AD. Graña et al. [28] obtained discriminant features from two scalar measures of DTI data and used support vector machine (SVM) as a classifier. From the view point of methodology, most of the studies focused on finding the microstructural differences using image analysis based on supervised machine learning algorithms. Although the analysis based on imaging is becoming an important research trend of AD studies, the technique is difficult to be applied in primary clinical settings because access to imaging equipment may be limited there.

Based on above-mentioned analysis, we proposed a computer-aided quick screening method for AD based on multiple neuropsychological rating scales. We especially used data mining technique to reduce the items of scales and lower the barriers to applying this method in primary clinical settings.

## 3. Preliminary

This section addresses some basic concepts needed for the remainder of the discussion. We introduce rough set theory first and then discuss genetic algorithm and Bayesian network, so as to set up a necessary context for describing our approach.

### 3.1. Rough Set Theory

Rough set theory [29–31] is a mathematical approach to deal with imprecision, vagueness, and uncertainty. The main difference between rough set theory and other mathematical tools for dealing with uncertain problems is that rough set theory does not need any prior information beyond the problem itself. Rough set theory is an important method for attribute reduction, where attributes that do not contribute to the classification of the given training data can be identified and removed.

Rough set theory is based on the establishment of equivalence classes within the given training data. All the data tuples forming an equivalence class are indiscernible; that is, the samples are identical with respect to the attributes describing the data. Formally, the indiscernibility relation is defined as follows:

(1) $$\begin{array}{c} \text{IND}\left(B\right)=\left\{\left(x,y\right)\in O^{2}\;|\;\forall a\in B,f\left(x,a\right)=f\left(y,a\right)\right\}, \end{array}$$

where *A* is a nonempty finite set of attributes and *O* is a nonempty finite set of objects. Given any *B*⊆*A*, relation IND(*B*) induces a partition of *O*, which is denoted by *O*/IND(*B*), where an element from *O*/IND(*B*) is called an equivalence class.

It is common that some classes cannot be distinguished by given real-world data in terms of the available attributes. Rough sets can be used to approximately or “roughly” define such classes. A rough set definition for a given class *X*, *X*⊆*O*, is approximately by two sets—a lower approximation of *X* and upper approximation of *X*. The lower approximation of *X* consists of all the data tuples that, based on the knowledge of the attributes, are certain to belong to *X* without ambiguity. The upper approximation of *X* consists of all the tuples that, based on the knowledge of the attributes, cannot be described as not belonging to *X*. So the lower and upper approximation of *X*, is defined, respectively, as

(2) $$\begin{array}{c} \underline{X}\left(B\right)=\left\{x\in U\;|\;{\left[x\right]}_{B}\subseteq X\right\},\\ \bar{X}\left(B\right)=\left\{x\in U\;|\;{\left[x\right]}_{B}\cap X\neq \Phi \right\}, \end{array}$$

where *x* ∈ *O* and the pair $\left(\underline{X}(B),\bar{X}(B)\right)$ is called the rough set with respect to *X*. The set ${BN}_{B}\left(x\right)=\bar{X}\left(B\right)-\underline{X}\left(B\right)$ is called the boundary region of *X*. Let [*x*]_*B*_ denote the equivalence class of relation IND(*B*) that contains element *x*.

An important concept in attributes reduction is dependency of attributes, which can be defined in the following way.

For *A* and *B*, *B* depends on *A* in degree *γ*_*B*_,

(3) $$\begin{array}{c} \gamma_{B}=\frac{\left|POS_{A}\left(B\right)\right|}{\left|O\right|}, \end{array}$$

(4) $$\begin{array}{c} POS_{A}\left(B\right)=\underset{X\in O/\text{IND}\left(B\right)}{⋃}{\underline{X}}_{A}, \end{array}$$

where *γ*_*B*_ = 1 means *B* depends totally on *A*. 0 < *γ*_*B*_ < 1 indicates *B* depends partially on *A*, and if *γ*_*B*_ = 0, *B* is totally independent on *A*.

Formally, a reduct is a subset of attributes which can fully characterize the knowledge in the database. Let RED(*B*) be the minimal subset of *A* and CORE(*B*) the set of attributes which cannot be eliminated and the intersection of all reducts. If the attribute in CORE(*B*) is removed, the ability to classify objects into the elementary classes of *B* will decrease:

(5) $$\begin{array}{c} \text{CORE}\left(B\right)=\underset{R_{i}\in \text{RED}\left(B\right)}{⋂},\;i=1,2,3,\ldots . \end{array}$$

### 3.2. Genetic Algorithm

The genetic algorithm (GA) [32–34] is an optimized algorithm based on the Darwinian principle of natural selection. It can be used with other data mining techniques for optimization and performance amelioration. The genetic algorithm process starts with the randomly generated and encoded initial population, which includes several hundreds or thousands of potential solutions to the problem. Each encoded individual in the population is called chromosome and each bit in the chromosome is called gene and has a value. The next step is called genetic operators. The most widespread genetic operators include selection, crossover, and mutation. Each chromosome in the population is evaluated by user-defined fitness function. The higher a chromosome's fitness value is, the more likely it is to produce offspring. In this way the overall fitness of the population is guaranteed to increase and those with weak fitness will be eliminated gradually. Crossover forms new chromosomes for the population by exchanging a fixed part between two chromosomes. The chromosomes most often used for crossover are those destined to be eliminated from the population. Mutation can be applied by randomly flipping bits (or attribute values) within a single chromosome to avoid the local optima. New offspring is reevaluated by fitness function to search the solution. The whole process is repeated until reaching the prespecified number of generations or the desired level of fitness.

### 3.3. Bayesian Network

Bayesian network is an acyclic directed graph for representing probabilistic relationships among a set of random variables [35]. Trained Bayesian networks can be used for classification. There are two key elements of a Bayesian network: (1) a directed acyclic graph (DAG) encoding the dependence relationships among a set of variables and (2) a probability table associating each node to its immediate parent nodes. Each node in the directed acyclic graph represents actual attributes given in the data. Each arc represents a probabilistic dependence. If an arc is drawn from a node *X* to a node *Y*, then *X* is a parent of *Y*, and *Y* is a descendant of *X* [36]. Bayesian network has one conditional probability table (CPT) for each node. The CPT for a node *Y* specifies the conditional distribution *P*(*Y*∣Parents(*Y*)), where Parents(*Y*) are the parents of *Y*. A node within the network can be selected as an “output” node, representing a class label attribute. There may be more than one output node. Given a set of variables, the network can be used to compute the probabilities of the presence of various classes, rather than return a single class label. There have been some works with applications using Bayesian network in diagnosis of AD [37, 38].

## 4. Methods

### 4.1. Overview

In this section, we present the formation process of AD diagnosis assisting model with the proposed hybrid intelligent method, which consists of two steps: in Step 1, use a genetic algorithm-rough sets (GA-RS) model to identify critical items, and in Step 2, use a Bayesian network to build a diagnosis assisting model of AD based on selected items.

In the first step, finding critical items from multiple neuropsychological scales is the problem of attributes reduction, which is also a classical problem in machine learning. Rough set theory is a useful attributes reduction method in machine learning. It can find the shortest or minimal reducts while keeping high-quality classification performance [39]. However, current rough set approaches to attributes reduction are inadequate to find optimal reductions as no perfect heuristic can guarantee optimality. Optimal attribute reduction has been proved to be a NP-hard problem [40]. So, stochastic approaches provide a promising attributes reduction mechanism, like genetic algorithm (GA). GA is an effective and robust method for solving both constrained and unconstrained multiparameter optimization problems that is based on natural selection. Many literatures have combined rough set theory and genetic algorithm for solving machine learning problems in a variety of domains [41–44]. We present a genetic algorithm for reduct set computation which is very fast and gives a good approximation in the AD field.

As exact causes and the mechanism of AD remain uncertain, there is currently no method to ensure the AD presence except for an autopsy. Clinicians can only make the diagnosis called “probable or possible AD” in clinical environment, especially in the early stage of this disease. The proposed approach applies Bayesian network that has strong reasoning ability in solving uncertain problems to build the decision-making model and predict the AD probability rather than offer a definitive diagnosis. So it is more conducive to the practical application of the proposed approach.

### 4.2. Attributes Reduction Based on Genetic Algorithm and Rough Set Theory

As mentioned above, GA-RS is used to identify critical items from a battery of rating scales. Each step of the algorithm is described as follows.

#### 4.2.1. Chromosome Representation

Because genetic algorithm cannot deal with data in solution space directly, we must represent them as binary strings of length *M* which is the number of the condition attributes by encoding. Binary encoding is simple and easy to operate. Each binary string is called a chromosome, in which “1” means that the corresponding attribute is selected and “0” means not. Attributes in* Core* should take “1”, and remain the same in the whole process of evolution, since genetic search starts from the* Core*.

#### 4.2.2. Fitness Function

Fitness function is a user-defined function which is used to measure each chromosome's optimization calculation in the groups. The fitness value of each chromosome represents suitability for the environment. In this paper, we expect the “best” chromosome could have the minimal length and the strongest classification performance as the algorithm proceeds. So the fitness function is defined as follows:

(6) Fx=β·fx+px=β·1−cardxcardC+cardPOSXDcardPOSCD,

where card(*x*) is the number of “1” in chromosome, which means the number of condition attributes contained by chromosome; card(*C*) is the length of chromosome, which is the total number of condition attributes; *f*(*x*) = 1 − card(*x*)/card(*C*) indicates the chromosome *x* that is not included in the proportion of condition attributes. *p*(*x*) indicates the distinction ability of attribute *x*.

#### 4.2.3. Selection Method

Select chromosomes based on their fitness values from the current population to produce offspring for the new population. Tournament selection is used, which means the higher the fitness value is, the higher probability of that chromosome is selected for reproduction. This step is repeated until the number of chromosomes selected is equal to the number of the population.

#### 4.2.4. Crossover and Mutation

One-point crossover method is used to reproduce with a probability of *P*_*c*_. In mutation process, we first select a chromosome to be mutated with probability *P*_*m*_ and then replace a single gene of the chromosome from “1” to “0” or from “0” to “1” randomly.

#### 4.2.5. Elitist Strategy

We take the elite strategy [45] to preserve the best individual of the fitness function value. Copy the individual of highest fitness value in the current generation to the next generation, unaltered.

The detail of the whole algorithm is as follows.

*Input*. Decision table *IS* = 〈*O*, *A*, *V*, *f*〉; *O* is a nonempty finite set of objects. *A* is a nonempty finite set of attributes: *A* = *C* ∪ *D*, *C* is the set of condition attributes, and *D* is the set of decision attributes. *V* = ⋃_*a*∈*A*_ *V*_*a*_; *V*_*a*_ is the set of values of attribute *a* ∈ *A*. *f* : *O* × *A* → *V* is an information function so that, for any *a* ∈ *A* and *x* ∈ *O*, *f*(*x*, *a*) ∈ *V*_*a*_.

*Output*. There is an attributes reduction *R* of decision table.

*Steps*

**Step 1 .**

Calculate the dependency *γ*_*c*_(*D*) between decision attributes set *D* and condition attributes set *C* by formula (3).

**Step 2 .**

Let Core(*C*) = *φ*, to get rid of each attribute *c* ∈ *C* one by one, if *γ*_*C*−*c*_ ≠ *γ*_*C*_, Core(*C*) = Core(*C*)∪{*c*} which means the core is Core(*C*); if *γ*_Core_(*D*) = *γ*_*C*_(*D*), then the core is minimal attributes reduction and if not, go to Step 3.

**Step 3 .**

Generate *m* binary strings with length *n* randomly, which can be seen as the initial population. *n* is the number of the condition attributes. “1” means that the corresponding attribute is present, and “0” indicates not. For attributes in core, corresponding position is “1” and for others, corresponding position is “1” or “0” randomly.

**Step 4 .**

Calculate the fitness value for each individual by formula (6) and select individuals by tournament selection.

**Step 5 .**

Perform crossover operation according to the crossover probability *P*_*c*_, using single-point crossover mode.

**Step 6 .**

Perform mutation operation according to the mutation probability *P*_*m*_. We basically bit mutation strategy while the corresponding bit of attributes in the* Core* does not change.

**Step 7 .**

Select the individuals with the best fitness values to be offspring of the current generation. This strategy is to guarantee the best chromosome could carry over to the next generation.

**Step 8 .**

Repeat the genetic operation until either one of the following conditions is satisfied: (1) the maximum number of generations is achieved or (2) the fitness value of the best individual for the present generation no longer changes during several successive generations.

**Step 9 .**

Convert the best individual to condition attribute and get the final result.

**Figure 2:**
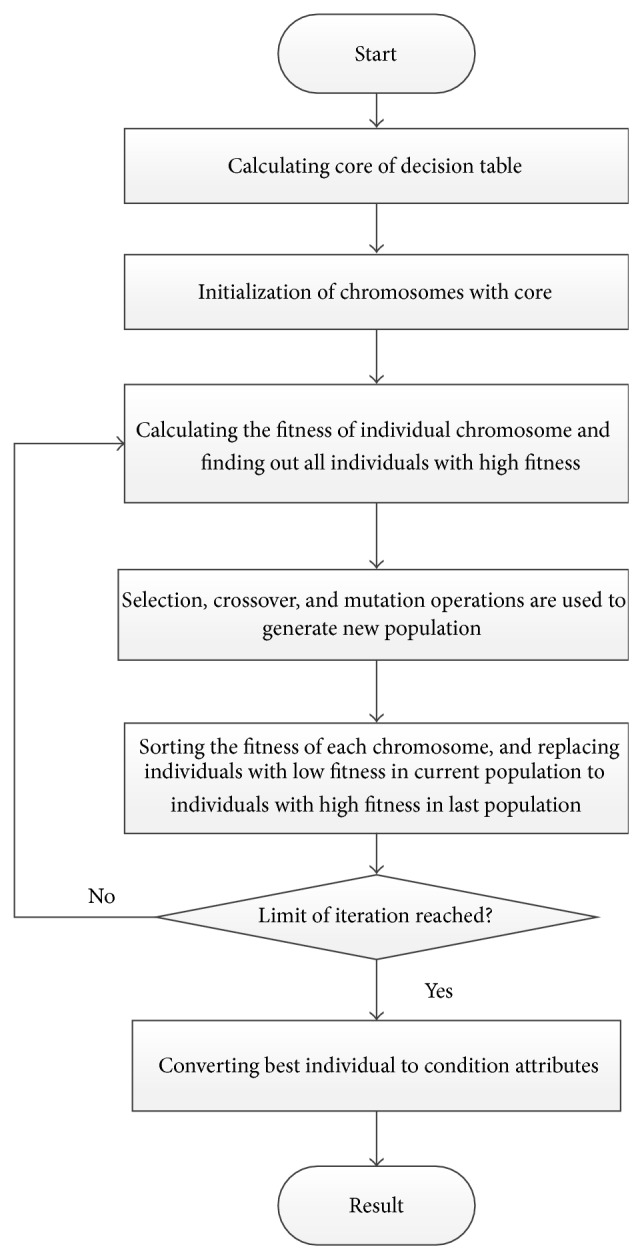
Detailed algorithm flow of GA-RS.

The whole computation steps are shown in Figure 2.

Parametric settings of genetic algorithm are as follows: population scale *N* = 1000, crossover ratio *P*_*c*_ = 0.5, mutation ratio *P*_*m*_ = 0.03, and the largest number of iterations is 500, just as demonstrated in Table 2.

The fitness function employed in this paper controls the chromosomes that evolve in the direction of the minimum reduction while keeping the classification performance: the higher the card(*x*) is, the smaller the *f*(*x*) is; the larger *p*(*x*), the more dependence between the condition attribute *C* and decision attribute *D*. This algorithm ensures the two requirements, so the result is the optimal solution of the problem.

In our approach, attributes reduction mentioned above is not the final goal but an intermediate process and core technology of AD diagnosis assisting for clinician in primary clinic. An uncertainty inference model for AD should be built after attributes reduction, which will be discussed in next section.

### 4.3. Bayesian Network Model for AD Diagnosis

Based on the above step, we attempt to construct the structural model for AD diagnosis. These selected items can be represented as input variables of the model. Since there is strong diagnostic uncertainty earlier in the disease process, an uncertainty inference model must be built. A popular modeling tool for complex uncertain domains is a Bayesian network.

## 5. Experiment and Results

### 5.1. Data Collection

The experimental data set is composed of 500 consecutive historical cases collected by the neurology department of a certain top hospital in China from 2009 to 2014. Each case is a series of scale scores belonging to one subject, and each subject has only one case. All neuropsychological tests were conducted by trained neuropsychologists and administered on the same day. The mean age of subjects is 74.4 (range, 51–92); 59.5 of the subjects' percent were female. These 500 historical cases have the following characteristics.

1. All these 11 neuropsychological rating scales are selected from a large number of scales by leading experts in neurology, including the MMSE, the MoCA, the CDR, the GDS, the ADL, the Word-List Learning, the figure copying, the new word discriminating, the trail making test, the similarity, and the perception. All these neuropsychological rating scales are commonly used instruments for screening cognitive or noncognitive impairment in the clinical diagnosis.
2. Each scale consists of a series of items. In total, there are 101 testing items in these scales. Some items are straightforward Q & A pattern, for instance, “What is the date?” Some others need the subject to do some actions, “Please read this and do what it says. (Show subjects the following words on the stimulus form: Close your eyes.)” Each of the tests scores points if it is answered correctly.

To ensure the correctness of diagnosis of each case, an expert panel group composed of three neuropsychologists was set up, and the diagnosis of each case was determined by the panel. The diagnosis of experts not only depended on an objective neuropsychological testing, but also on the history-taking from the patient and a knowledgeable informant. Their diagnosis was regarded as the gold standard. In the current study, the diagnosis of cases could be divided into three types: patients with AD, patients meeting criteria for mild cognitive impairment [46] (called MCI for short, which is regarded as the predementia stage of AD), and the elderly subjects with normal cognition, in which, the number of each type is 33.5%, 37.7%, and 28.8%, respectively.

Parts of cases are given in Table 3. In the table, each column is one testing item of scales, for instance, Time Orientation, Place Orientation and Repetition belong to the MMSE while Visuospatial Skills belongs to the MoCA. They are regarded as the condition attributes. The last column, Result, is the decision attribute (the diagnosis of each patient).

### 5.2. Experimental Design and Results

To verify the feasibility and validity of the proposed approach, the performance of proposed approach can be measured by the following evaluations: (1) reduction ratio on testing duration and reduction ratio on quantity of items; (2) comparison with multiple classifiers; (3) comparison of classification accuracy before and after reduction; (4) the performance of classification compared with two existing cognitive screening scales.

We applied two evaluation methods to prove the reliability of our experimental results, one was 10-fold cross-validation, and the other was 0.632 bootstrap. Obtain computing results by averaging after executing 10 times. Recall rate, precision rate, and accuracy were selected as the performance evaluation metrics. The obtained results of the experiment are to be presented and discussed in the next section.

### 5.3. Reduction Result

After attributes reduction, 10 items were selected finally, which are listed in Table 4.

Some items represent one test, such as Figure_Copy, Figure_Short_Memory, Figure_Delay_Memory, Word_Delay_Recall, and Reading_Comprehension, while others are a set of several tests, which cannot be separated (because these tests significantly correlate with one another and must be performed together), such as Visuospatial_Execution, IADL, Naming, Attention, and Word_AVG.

#### 5.3.1. Reduction Ratio

We used reduction ratio including the reduction ratio of testing duration and the reduction ratio of quantity of items as measurable metrics. Assume that the number of condition attributes before and after reduction is *m* and *n*, respectively. The reduction ratio *r* is defined:

(7) $$\begin{array}{c} r=\frac{\left(m-n\right)}{m}\times 100\%. \end{array}$$

Before reduction, the number of items is 34, while only 10 items left after reduction using the proposed method, so we can conclude that the reduction ratio is 70.59%. The experimental results indicate that using GA-RS to select subset can reduce items dramatically.

Similarity, the reduction ratio of testing duration can also be calculated using formula (7). In clinical practice, the duration of finishing these scales varies a lot, which depends on the subject's state of cognitive impairment. According to [47, 48], the performance time for the MMSE and the MoCA is 13.4 minutes and 14.8 minutes on average, respectively. Based on the past experience, a skilled clinician administers the scale for more than one hour to complete all the 11 scales mentioned above. By using the proposed model, clinicians do not have to fiish all the scales but only need to complete the selected testing items. Hence the test duration is reduced greatly and ranges from 12 to 15 minutes with a mean time of 13.5 minutes and a standard deviation of 2.3 minutes.

#### 5.3.2. Comparison with Multiple Classifiers

**Figure 3:**
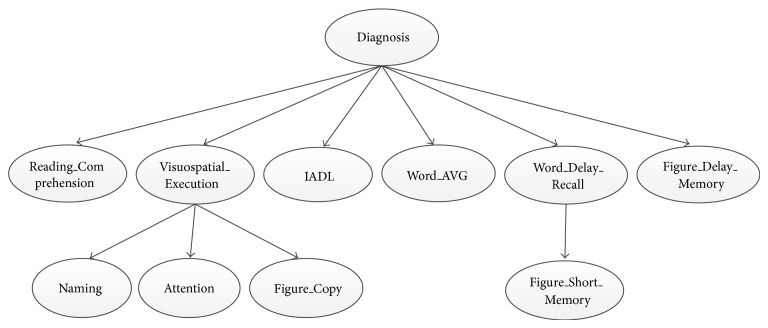
AD-related diagnosis assisting model.

The constructed Bayesian network structure is presented in Figure 3.

We compared some common used classifiers with Bayesian network in order to select the well-performed classifier. All these classifiers had the same input items (the items selected by the above step). The result of comparison is as shown in Table 5.

From Table 5, we could see that the Bayesian network performed best in the four classifiers. Then, in order to further prove the effectiveness of results, we compared the four groups using Friedman test to see if a significant difference emerged (Table 6). Table 6 shows the mean rank for each classifier and Table 7 shows the result of the Friedman test.

In Table 7, *P* = 0.001 < 0.05, there was a significant difference between these four classifiers. However, the cross-validation estimate of prediction error may lead to a high variability in results. In order to validate the result further, 0.632 bootstrap was used to evaluate the performance, as shown in Table 8.

Similar result came from Table 8 when compared with Table 5. It suggested that Bayesian network performed better than other three classifiers. And Friedman test was also performed, as shown in Tables 9 and 10. We found that there existed a significant difference between the four groups as well. Because of low variance with only moderate bias, the result got by 0.632 bootstrap was selected as the final result.

#### 5.3.3. Classification Performance before and after Reduction

In order to evaluate the validity of attributes reduction, we used Bayesian network algorithm to compute the classification performance before and after attributes reduction, respectively, and to check whether or not the classification performance had changed.

Each subject had been given the probability of each classification. The highest probability was regarded as the diagnosis of the model.  Table 11 presents a summary of the classification results before and after reduction.

From the variance of recall rate and precision rate after attributes reduction as shown in Table 11, we found that the recall rate and precision rate of each group decreased a little, but less than 3.05%. We analyzed the result data using Wilcoxon Signed-Rank Test. The calculated *P* value was 0.853 and larger than 0.05, so the null hypothesis was true, which means that there was no significant statistical difference between these two methods. In conclusion, the comparative experimental results indicated that the proposed method could find the shortest or minimal reducts while keeping high-quality classification performance.

#### 5.3.4. Comparison with Comprehensive Cognitive Screening Scales

Comprehensive cognitive screening scales measure all important aspects of cognitive function, such as memory, language, visuospatial skills, attention, and executive function. The most commonly used comprehensive cognitive screening scales include the MMSE and the MoCA. Our computer-aided model also covers multiple aspects of cognitive screening, so the comparison of recall rate and precision rate between our model and these two scales is needed based on the same dataset in order to evaluate the validity of our model.

The MMSE is a questionnaire test that is used to screen cognitive impairment. The total score is 30. If the score is greater than or equal to 27 points, it means the subject has a normal cognition. Below 27 points, scores can be divided into several stages: severe cognitive impairment (≤9 points), moderate cognitive impairment (10–18 points), and mild (19–24 points) cognitive impairment [49]. The MoCA is also a one-page 30-point test developed as a brief cognitive screening tool to detect mild-moderate cognitive impairment. The suggested cut-off score on MoCA is 26, which yielded the best balance between sensitivity and specificity for the MCI and AD groups. We applied these criteria to our dataset, and the result is showed in Table 12.

From Table 12, we found that the MMSE did not perform well as a screening instrument for MCI due to the lack of sensitivity to MCI [50]. Some researchers believe the low sensitivity of the MMSE comes from the emphasis placed on language items and a paucity of visuospatial items [51]. However, visuospatial skills and executive function had been retained through our attributes reduction algorithm. So our model performed much better than the MMSE on detecting MCI, which is a very significant stage of AD. In general, our model is a more effective tool for identifying different stages of AD than the MMSE.

The MoCA is designed for the detection of MCI; that is to say, it is developed to screen patients who has cognitive impairment complaints but still performed in the normal range on the MMSE. The MoCA is more sensitive on detecting MCI than the MMSE, because the MoCA focuses more on tasks of frontal executive functioning and attention. Our model retained these key parts of the MoCA, so its performance on detecting MCI was close to that of the MoCA. Above all, our model was advantageous when identifying multiple transition points between different stages of AD, and it was not only designed for screening MCI. Compared with the MoCA, our model had distinct advance on differentiating normal and AD while almost keeping the sensitivity of detecting MCI. This was more helpful for primary clinicians to take target care and therapies.

We also performed Friedman test on these three groups and the actual result of the Friedman test is shown in Tables 13 and 14. From the result, we can see that there is an overall statistically significant difference between the mean ranks of these three groups.

## 6. Discussion

This study proposed a computer-aided diagnosis model of AD applied in primary clinics. In order to solve especially the problem that cognitive screening based on multiple neuropsychological scales was time consuming, GA-RS algorithm was used to identify the most related items from numerous rating scales while ensuring a satisfactory accuracy of classification. The experimental results validated the effectiveness of the proposed approach.

The benefits of this study are listed as follows.

- The proposed approach is suitable to be applied in the primary clinics, because the clinician's day is labor-intensive, and the mean time of clinical interviewing is limited for each patient. Clinicians are eager to have a tool for the screening of AD without spending too much time. The proposed approach reduces the testing time for each patient while keeping classification accuracy. Hence, such computer-aided approach is applicable in clinical practice.
- As an important merit of the proposed approach, it is a computer-aided diagnostic tool based on multiple neuropsychological rating scales, rather than neuroimaging, or biomarker. To the best of our knowledge, there exist few reports providing a computer-aided diagnosis method that is completely based on multiple neuropsychological rating scales. Thus our approach is suitable to be popularized in the primary clinics which have no advanced imaging and biological molecular equipment.
- It is estimated by the specialists that there are 68 relevant AD scales in the world [52], most of which have established the normative data and interpretation of scores in different countries. However, there have been seldom studies on how to employ so many rating scales to give a comprehensive diagnosis. For instance, if a patient “MMSE = 27”, “MoCA = 23”, and “ADL = 26,” then what is the comprehensive status of the patient? It is relatively difficult for young general practitioners to make diagnosis in primary clinics. Our approach provides a new thought to solve this problem in hopes of supplementing the research in this field.
- The proposed approach is based on neuropsychological rating scales. Any disease that has no specific golden criteria and needs a long test by rating scales can try this method.

It should also be mentioned that there remain some limits to the approach proposed in this paper.

First of all, the chosen data might be bias, as all the cases of the study were collected only in one hospital. Secondly, there are some other types of dementia, such as Lewy body dementia and vascular dementia, which are difficult to be distinguished from AD for young practitioners. The cases of these diseases are not included in our dataset, so clinicians must differentiate diagnostic methods for these diseases when using our model. Thirdly, the classification performance of Bayesian Network does not perform as well as expected, more machine learning algorithm can be tried to improve the classification performance. Finally, the number of subjects is still limited and more subjects are necessary in order to generalize the results to a larger population.

## 7. Conclusion and Future Work

The increasing aging population has led to a high increase in the prevalence of AD. Due to the fact that targeted care and therapies may slow down the progression of disease, the identification of different stages of AD is very important. In this paper, we proposed a computer-aided diagnosis method for AD based on analyzing the practical scores of rating scales. We especially identified the most discriminative items based on rough set theory and genetic algorithm. The selected items cover multiple cognitive domains and can be administered generally within 15 minutes. So it is user-friendly and is quickly administered, it may be appropriate use in primary clinics where assessment time is often limited. By comparing the classification performance, the result showed that the approach can effectively reduce the representation space of the attributes whilst hardly decreasing classification precision. The data also indicated that it has satisfactory reliability for both MCI and AD comparing with other existed cognitive screening scales.

Without doubt, opportunities for future research are abundant. First, we plan to further evaluate the built model with a perspective study in a real clinical setting. Second, more rating scales for specific dementias are going to be involved in the training set data and more comprehensive model for senile dementia will be built in the future work. Based on above work, a “three-level medical service network” for AD is going to be built in the near future and different computer-aided diagnosis tools for each level hospital will be developed; for example, the simple cognitive screening tool helps clinicians in primary clinics to judge whether patients suffer from cognitive impairment; the advanced cognitive assessment tool helps clinicians in second class hospitals to estimate the severity of cognitive impairment; the comprehensive assisted diagnosis tool is designed for clinicians in top hospitals to differentiate the types of dementia. The setup of such network will improve diagnosis accuracy of AD greatly and reduce the burden on public health care resource.

## Figures and Tables

**Table 1:** Minimental state examination (MMSE).

Orientation	Year Month Day Date Time	___/5
	Country Town District Hospital Ward	___/5

Registration	Examiner names 3 objects (e.g. apple, table, and penny). Patient asked to repeat (1 point for each correct answer). THEN patient to learn the 3 names repeating until correct.	___/3

Attention and calculation	Subtract 7 from 100 and then repeat from result. Continue 5 times: 100 93 86 79 65 Alternative: spell “WORLD” backwards-“DLROW”	___/5

Recall	Ask for names of 3 objects learned earlier.	___/3

Language	Name a pencil and watch	___/2
	Repeat “No fits, ands, or buts”	___/1
	Give a 3-stage command. Score 1 for each stage. E.g., “Place index finger of right hand on your nose and then on your left ear”	___/3
	Ask patient to read and obey a written command on a piece of paper stating “Close your eyes”	___/1
	Ask patient to write a sentence. Score if it is sensible and has a subject and a verb	___/1

Copying	Ask the patient to copy a pair of intersecting pentagons: * * 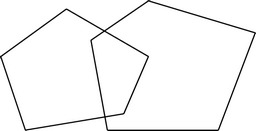	___/1

	* *Total	___/30

**Table 2:** Parameter settings of GA.

Population size	1000
Number of generations	500
Initialization method	Binary method
Percentage of elite	0.2
Selection method	Tournament selection
Crossover method	Uniform crossover
Crossover ratio	0.5
Mutation method	Single-point mutation
Mutation ratio	0.03

**Table 3:** Part of AD dataset.

Fact	Time orientation	Place orientation	Repetition	Visuospatial skills	⋯	Result
1	5	5	1	5	⋯	Normal
2	4	5	1	5	⋯	Normal
3	4	5	1	3	⋯	MCI
4	1	4	0	3	⋯	AD
⋮	⋮	⋮	⋮	⋮	⋮	⋮
500	0	0	1	1	⋯	AD

**Table 4:** Reduction results by GA-RS.

Result	Source
Reading_Comprehension	MMSE
Visuospatial_Execution	MoCA
Naming	MoCA
Attention	MoCA
Figure_Copy	Figure copying test
Figure_Short_Memory	Figure copying test
Figure_Delay_Memory	Figure copying test
IADL	ADL
Word_Delay_Recall	Word-List Learning test
Word_AVG	Word-List Learning test

**Table 5:** The comparison of classifiers by 10-fold validation.

	Normal		MCI		AD		ACC%
	*R* (%)	*P* (%)	*R* (%)	*P* (%)	*R* (%)	*P* (%)	
BN	85.41	87.23	78.95	73.17	90.7	95.12	85.27
NN	77.27	79.07	69.05	64.44	86.05	90.24	77.52
C4.5	81.82	63.16	42.86	48.65	72.09	88.57	65.89
SMO	86.36	74.51	64.29	77.14	93.02	93.02	81.35

*R*: recall rate; *P*: precision rate; ACC: accuracy; BN: Bayesian network; NN: neural network.

**Table 6:** Ranks in Friedman tests.

	Mean rank
Bayesian network	2.58
Decision tree (C4.5)	2.27
Neural network	2.50
SMO	2.66

**Table 7:** Test statistics in Friedman tests.

Chi-square	15.789
df	3
Asymp.Sig.	0.001

**Table 8:** The comparison of classifier by 0.632 bootstrap.

	Normal		MCI		AD		ACC%
	*R* (%)	*P* (%)	*R* (%)	*P* (%)	*R* (%)	*P* (%)	
BN	84.55	84.91	75	76.39	91.94	89.81	83.73
NN	78.99	71.76	60.63	67.68	91.55	91.98	76.94
C4.5	71.90	66.67	46.45	50.26	79.15	80.29	66.24
SMO	84.71	79.46	63.03	69.63	89.57	87.91	79.61

*R*: recall rate; *P*: precision rate; ACC: accuracy; BN: Bayesian network; NN: neural network.

**Table 9:** Ranks in Friedman tests.

	Mean rank
Bayesian network	2.59
C4.5	2.30
Neural network	2.54
SMO	2.57

**Table 10:** Test statistics in Friedman tests.

Chi-square	48.694
df	3
Asymp.Sig.	0.000

**Table 11:** Comparison of the classification performance before and after attributes reduction by Bayesian network.

	Normal		MCI		AD		ACC%
	*R* (%)	*P* (%)	*R* (%)	*P* (%)	*R* (%)	*P* (%)	
Before	86.36	84.44	76.19	76.19	90.7	92.86	84.50
After	84.55	84.91	75	76.39	91.94	89.81	83.73

*R*: recall rate; *P*: precision rate; ACC: accuracy.

**Table 12:** Comparison with the MMSE and the MoCA.

	Normal		MCI		AD		ACC%
	*R* (%)	*P* (%)	*R* (%)	*P* (%)	*R* (%)	*P* (%)	
MMSE	93.88	67.65	45.83	61.11	84.29	93.65	74.29
MoCA	68.89	86.11	81.82	60.00	80.39	93.18	77.14
Model	84.55	84.91	75.00	76.39	91.94	89.81	83.57

*R*: recall rate; *P*: precision rate; ACC: accuracy.

**Table 13:** Ranks in Friedman tests.

	Mean rank
MMSE	1.94
MoCA	1.98
Model	2.08

**Table 14:** Test statistics in Friedman tests.

Chi-square	3.756
df	2
Asymp.Sig.	0.043
